# A Review of Biomaterials and Associated Performance Metrics Analysis in Pre-Clinical Finite Element Model and in Implementation Stages for Total Hip Implant System

**DOI:** 10.3390/polym14204308

**Published:** 2022-10-13

**Authors:** Md Mohiuddin Soliman, Muhammad E. H. Chowdhury, Mohammad Tariqul Islam, Farayi Musharavati, Mohammad Nabil, Muhammad Hafizh, Amith Khandakar, Sakib Mahmud, Erfan Zal Nezhad, Md Nazmul Islam Shuzan, Farhan Fuad Abir

**Affiliations:** 1 Department of Electrical, Electronic & Systems Engineering, Universiti Kebangsaan Malaysia, Bangi 43600, Selangor, Malaysia; 2 Department of Electrical Engineering, Qatar University, Doha 2713, Qatar; 3 Centre for Advanced Electronic and Communication Engineering, Department of Electrical, Electronic and Systems Engineering, Faculty of Engineering & Built Environment, Universiti Kebangsaan Malaysia (UKM), Bangi 43600, Selangor, Malaysia; 4 Department of Mechanical & Industrial Engineering, Qatar University, Doha 2713, Qatar; 5 Department of Biomedical Engineering, University of Texas at San Antonio, San Antonio, TX 78249, USA

**Keywords:** total hip implant, finite element analysis, stress distribution, micromotion, wear, biocompatibility

## Abstract

Total hip replacement (THR) is a common orthopedic surgery technique that helps thousands of individuals to live normal lives each year. A hip replacement replaces the shattered cartilage and bone with an implant. Most hip implants fail after 10–15 years. The material selection for the total hip implant systems is a major research field since it affects the mechanical and clinical performance of it. Stress shielding due to excessive contact stress, implant dislocation due to a large deformation, aseptic implant loosening due to the particle propagation of wear debris, decreased bone remodeling density due to the stress shielding, and adverse tissue responses due to material wear debris all contribute to the failure of hip implants. Recent research shows that pre-clinical computational finite element analysis (FEA) can be used to estimate four mechanical performance parameters of hip implants which are connected with distinct biomaterials: von Mises stress and deformation, micromotion, wear estimates, and implant fatigue. In vitro, in vivo, and clinical stages are utilized to determine the hip implant biocompatibility and the unfavorable local tissue reactions to different biomaterials during the implementation phase. This research summarizes and analyses the performance of the different biomaterials that are employed in total hip implant systems in the pre-clinical stage using FEA, as well as their performances in in vitro, in vivo, and in clinical studies, which will help researchers in gaining a better understanding of the prospects and challenges in this field.

## 1. Introduction

**Figure 1:**
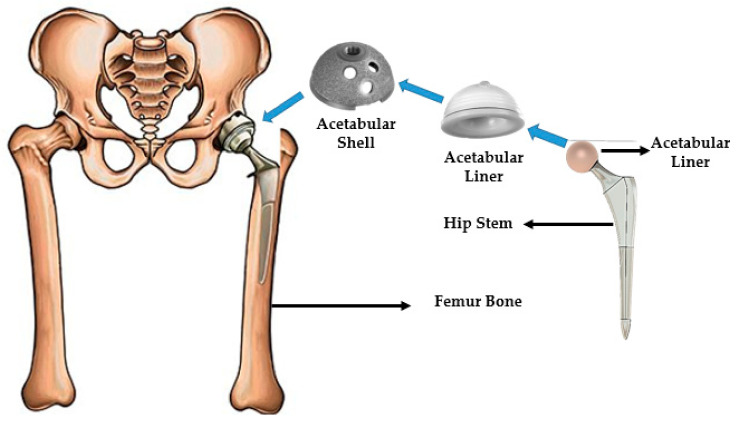
Total hip implant system.

The hip joint is very significant in the human body since it supports the entire body’s weight during dynamic load conditions and assures that the femoral bone is united with the pelvis. Hip mobility can be impaired by rheumatoid arthritis, accidents, and other unfortunate occurrences. Millions of people worldwide suffer from hip joint injuries each year [1,2,3,4]. Gender, age, physical activity, and height increase the potential of osteoporosis [2,3,4]. Osteoporosis is defined as a bone wear that occur with age advancement, which make the patients highly vulnerable to bone fractures. The hip implant is an excellent therapeutic option for extreme osteoarthritis, the crippling symptoms of rheumatoid arthritis, birth deformities, and certain post-traumatic states. The total hip replacement (THR) with an implant is a well-established procedure, and it is has been most frequently performed joint replacement in orthopedic surgery over the previous two decades [5]. For nearly half a century, the field of hip implants has significantly advanced the mobility and quality of life of the patients from both athletic and non-athletic backgrounds. Charnley [5] and Watson Farrar [6] began the modern era of successful hip replacements in the mid-1960s. From 2012 to 2018, the American Joint Replacement Registry (AJRR) recorded a total of 602,582 hip replacement processes that were performed where 80.5% of them were first-time hip replacements, 9.1% of them were revision THR, 8.2% of them were partial hip replacements, and 2.2% of them were THRs [7]. Figure 1 depicts a typical hip replacement system in which the femoral head and acetabulum are replaced by an artificial hip implant or a prosthesis. An artificial hip implant involves a metal stem, which is known as the femoral component, and a cup, which is known as the acetabular component.

Although the THR is gaining popularity among surgeons, biomechanical engineers and physicists are concerned about the post-surgical degeneration of the prosthetic joints. The ideal material selection for the hip implants must satisfy certain requirements, such as a proper stress distribution, a long fatigue life, biocompatibility, a high degree of strength, a high degree of corrosion resistance, a low cost, and malleability compatible with dynamic human movement [8]. To pick the ideal material for a hip implant that meets these criteria, it is required to test the performance metrics of a hip implant at multiple phases, namely those which are pre-clinical, in vitro, in vivo, and clinical. In the field of hip implants, the Finite Element Method (FEM) has proven its efficacy and capabilities in addressing the mechanical response of the implant in a cost-effective and pre-clinical environment [9]. According to the studies [8,9,10,11], computational finite element analysis (FEA) can be employed in the pre-clinical phase to evaluate four mechanical performance factors, such as von Mises stress and deformation [8], micromotion [10], wear estimation [9], and fatigue life estimation [11]. During in vitro, in vivo, and clinical phases, the biocompatibility and adverse local tissue reactions of the hip implant system are evaluated. The important mechanical features for good bone remodeling at the bone–implant interface are enough to transfer the load from the hip implant to the bone and evenly distribute the stress over the hip implant. This investigation is carried out through the use of an FEA method. In addition, sufficient micromotion at the bone–implant interface is required for adequate bone re-modelling and implant integration to occur. The literature reveals that, as a post-surgical problem, wear debris propagation is the leading cause of a hip implant component loosening, which can result in a premature implant failure and its revision [12,13,14]. Osteoporosis is significantly linked to implant degeneration, which is induced by polyethylene wear particles as a result of prolonged articulated movement between the acetabular cup and the metal femoral head [15]. Additionally, wear debris is formed by the various material combination hip implant systems, including those that are made of metal-on-metal (MoM), metal-on-ceramic (MoC), ceramic-on-ceramic (CoC), and ceramic-on-polyethene (CoP) combinations. This is a leading source of the adverse tissue reactions that occur in the human body [16]. Given that the anticipated functional life of an implant depends on its material qualities and other design criteria, a computational analysis using FEA can be used to determine the functional fatigue life of it. Therefore, it makes sense to use computational FEM analysis in the pre-clinical phase to assess the performance parameters. Researchers are enhancing the design and materials of hip implants by using computational FEM analyses to suit their research goals. Before mass production of them can occur, in vitro, in vivo, and clinical research must be undertaken to validate the adverse tissue reaction findings as the results of a computational FEM analysis can only demonstrate the mechanical performance of different biomaterial hip implants. This article explains and assesses the properties of the biomaterials that are used in the whole hip implant systems, as well as the performance metrics that are derived during the pre-clinical, in vitro, in vivo, and clinical stages.

The remaining part of the article is structured as follows: the mechanical and physical properties of the biomaterials of a hip implant and the introduction of the associated hip implant components are discussed in Section 2. The performance parameters of the hip implant with different biomaterials which are estimated in the pre-clinical stage are discussed in Section 3, while in in vivo, in vitro, and clinical stages are discussed in Section 4. Section 5 presents a comprehensive assessment of the major factors of the hip implants with different biomaterials to identify the research problems with them. Finally, Section 6 contains a summary of the article.

## 2. Biomaterials for Total Hip Implant System

In vivo studies have been performed for a long time to figure out how to choose the best material for the hip implant parts. This is because the human body is an asymmetrically hostile environment. To reduce the risk of a failure occurring and increase the patient’s safety, the mechanical performance of the hip implant should be optimized concerning the tensile stress and Young’s modulus. Because the implant must survive in the human body, the implant material must also be biocompatible, non-toxic, chemically stable, and wear resistant. This section will explore the executive summary of the existing materials that are associated with the Total Hip Implant (THI) system. The first subsection provides an overview of mechanical factors relating to the hip implant biomaterials. The second section discusses the physical and chemical properties of the biomaterials that are used in hip implants.

### 2.1. Important Parameters for Hip Implant Materials

The mechanical and physical properties of a hip implant’s biomaterial indicate the implant’s performance against the high loading values of human activities.

#### 2.1.1. Young’s Modulus

Young’s modulus (modulus of elasticity) is a material characteristic parameter that defines the stiffness of a material, and it is one of the most significant parameter to consider in selecting the hip implant biomaterials. The mechanical and physical properties of the biomaterial in a hip implant determine how well it will work under the high loads that come with the performance of human activities. Young’s modulus is a material property that describes how stiff a material is, and a higher modulus of elasticity means that the material is stiffer. Young’s modulus is the ratio of stress-to-strain in the area where the material is deforming elastically, as defined by Equation (1) [17].

(1) $$E=\frac{Stress}{Strain}=\frac{\frac{F}{A}}{\frac{\Delta L}{L_{0}}}=\frac{FL_{0}}{A\Delta L}$$

where *E* is Young’s modulus in Pascal, *F* is the force that is exerted on an object that is under tension, *A* is the area of the cross-section that is perpendicular to the applied force, ΔL is the change in the length of the object, and $L_{0}$ is the original length of the object.

#### 2.1.2. Fatigue Crack

The term “fatigue crack” is used in material science research to characterize the occurrence and propagation of fractures in a material because of cyclic stress. The crack expansion rate $\frac{da}{dN}$ can be used to predict the material’s resistance to a fatigue crack. When the applied loading stress on the material is greater than the tensile strength is, then the material will fracture. The crack expansion rate is related to the strength of the material (*K*) and Young’s modulus (*E*), as shown in Equation (2) [17].

(2) $$\frac{da}{dN}=C\Delta K^{m}=C_{a}{\left(\frac{K}{E}\right)}^{3.6}\;\;\;$$

where $\frac{da}{dN}$ is the crack growth rate [mm/cycle], *C*, *Ca*, and *m* are the experimental constants, ΔK=Δσ is the stress intensity factor, and E is Young’s modulus. The critical tensile stress of the material can be calculated using Equation (3).

(3) $$\sigma_{crit}=\sqrt{\frac{E\gamma }{\pi a}}\;\;$$

where $\sigma_{crit}$ is the critical tensile stress, γ is the energy per unit area of the new surfaces, and a is the crack length for a surface crack.

#### 2.1.3. Stress Shielding

Mismatching the implant material mechanical characteristics with the bone can cause stress shielding to occur, incorrect micromotion at the bone–implant contact area, and hip loosening [18]. Greater implant stiffness reduces the amount of stress at the implant–bone interface due to there being differences in Young’s modulus. A moderate Young’s modulus mismatch between the implant and the bone reduces the amount of stress shielding and bone deterioration that occurs at the implant–bone contact area. Low Young’s modulus alloys have a lower critical tensile strength, which increases the fatigue cracking risk according to Equation (3). Considering these factors, the ideal material selection for the hip implants must undergo a pre-clinical analysis via FEA to identify the implant’s stress, strain, deformation, wear estimation, and fatigue life under the most frequent human activities.

#### 2.1.4. Wear Behavior

Wear is described as a progressive surface degradation phenomenon that is characterized by the removal of material in the shape of microscopic particles from the body, which primarily occurs by a mechanical process [16]. Five numerical models are applied to estimate the wear rate [19]. According to the early-stage wear estimation formula, which is known as Archard’s wear law and is described in Equation (4), the wear rate primarily depends on the coefficient of wear of the joint materials [20].

(4) $$dV=\Delta AdH=K_{w}\times \sigma \times \Delta A\times dS=K_{w}\times \sigma \times \Delta A\times V_{sd}\left(t\right)\times dt\;$$

and here,

$$dH=K_{w}\times \sigma \times dS$$

where dH is the wear depth (mm/cycle), σ is the contact pressure in contact joint surface, dS is sliding distance, and ΔA is the contact area (mm^2^). The wear coefficient $K_{w}$ is the wear constant for different joint material combinations. Hence, the wear volume can be determined by integrating Equation (4) over the full human gait cycle time (*t*) using the following Equation (5).

(5) $$\;V\left(t\right)=K_{w}\times \Delta A\int_{0}^{t}\sigma \left(t\right)\times V_{sd}\left(t\right)\times dt\;\;$$

Hence, according to the following formula, the wear rate is completely dependent on the wear coefficients. According to results that are stated in [16,20], a lower wear coefficient value was obtained for stiffer materials. Although a stiffer material combination for the joints reduced the rate of the wear, biocompatibility must still be maintained. In Section 3.2, the specifics of the wear rate estimation by the FEA for various material combinations are provided. In addition, Section 4 elaborates on the biocompatibility and tissue response of various biomaterials in, in vitro, in vivo, and clinical research.

### 2.2. Material Used in Total Hip Implant System

According to the existing established implant model that is obtained from famous orthopedic implant manufacturing firms, the stem of the hip implants are made from titanium, chrome, zirconium, stainless steel, and alumina alloys [21]. Commercially available pure titanium and hydroxyapatite (HA) are used for the porous shape coating on the hip implant’s body segment. A highly refined Co-Cr-Mo alloy, an Al_2_O_3_-sintered ceramic composite, or stabilized ZrO_2_ with Al_2_O_3_ and yttrium oxide additives are frequently utilized as materials in the acetabular head fabrication. Additionally, polyether-ether-ketone (PEEK) and ultra-high molecular weight polyethene (UHMWPE) have been utilized as effective biomaterials for the acetabular liner part because they have good mechanical features. The following section outlines the mechanical and chemical properties of the biomaterials that are associated with the hip implant system.

#### 2.2.1. Metallic Alloys

According to the literature review that is provided below, titanium and cobalt alloys were used to construct the hip implant system. In this subsection, the material properties of the commercially available metallic alloys that are utilized in hip implant systems are illustrated.

#### 2.2.2. Titanium Alloys

Titanium alloys and pure titanium are appropriate for their use in hip implants due to their biocompatibility, low density, and osteointegration (bone connection) properties [17]. The most common titanium alloys are α, β, and α + β, which are characterized by their microstructure and phase composition [22]. α-type titanium alloys include Al, O, N, and C, while β-type alloys include V, Nb, Ta, Mo (isomorphous), Fe, W, Cr, Ni, Si, Co, Mn, and H (eutectoid) [22]. Table 1 shows the biomaterial alloys that are used in commercial hip implants [17,23,24,25]. α + β titanium alloys have a Young’s modulus of 110–120 Gpa, which is higher than it is for bone. Several studies are examining the factors that affect the titanium alloy Young’s modulus. The body-centered cubic microstructure phase has a lower Young’s modulus than the hexagonal one does [26].

**Figure 2:**
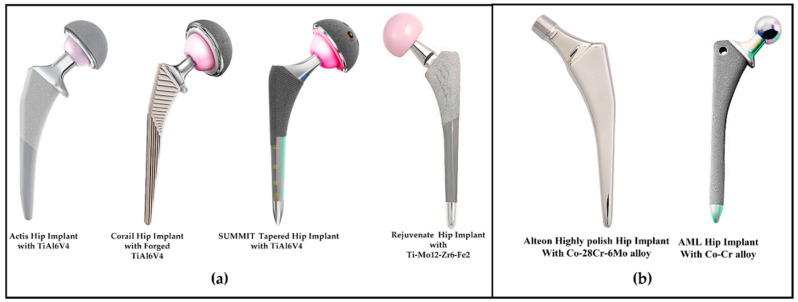
Several clinically successful total hip implants with (**a**) titanium alloys [33,34], and (**b**) cobalt alloys [33,34].

The martensite phase [27,28] and thermal-mechanical process [29] in the titanium alloys increase the strength of the microstructure. If the omega phase is increased compared to the β one, then the martensitic phase increases the Young’s modulus [30]. Lowering the Young’s modulus reduces the static strength, such as the tensile strength, which is important when choosing a hip implant material. High-pressure torsion, accumulating roll-bonding, and equal-channel angular pressing [31] can boost the tensile strength of the lower-Young’s-modulus materials such as Ti-29Nb-13Ta-4.6Zr [32]. Ageing improves the dynamic strength, such as the fatigue strength, in the lower-Young’s-modulus materials, such as Ti-29Nb-13Ta-4.6Zr, wherein, the fatigue strength can be increased to 600 Mpa from 450 Mpa [32]. Ceramics improve the fatigue strength by lowering the Young’s modulus [32]. Well-known orthopedic businesses make hip implants using titanium-based alloys like Ti-6Al-4V and TiMo_12_Zr_6_Fe_2_ [33,34]. Figure 2 shows the hip implant systems with titanium hip stems that were manufactured by renowned manufacturers. The first three hip implant systems, namely Actis, Corail, and Summit, have achieved clinical success [33], however, the Rejuvenate Hip System which features a dual-modular-type femoral stem (a femoral stem combined with an exchangeable neck) has demonstrated that it has a higher revision rate [35]. In summary, although titanium alloys seem to be promising for hip implants, further computational research using an FEM analysis is required to validate the mechanical performances of them, such as their amount of stress, strain, and deformation.

#### 2.2.3. Cobalt Alloys

Before the titanium, cobalt-chromium alloys were utilized in hip implants. Hip implants were commercially produced using cobalt-chromium-nickel-molybdenum alloys such as Co-28Cr-6Mo [33]. Figure 2 shows the effective hip implant systems with cobalt metal hip stems. In Co-28Cr-6Mo alloys, the composite ingredients are 58.9–69.5% Co, 27.0–30% Cr, 5.0–7.0% Mo, and a little amount of Mn, Si, Ni, Fe, and C [16]. Using a forging grain size of fewer than 10 microns can be performed, while the casting process can provide grain sizes between 30 and 1000 microns. Cobalt-chromium alloys offer a high yield and an ultimate strength because of their multiphase structure and carbide precipitation. Annealing can increase the tensile strength, fatigue, and elongation of the cobalt-chromium alloy [36].

**Figure 3:**
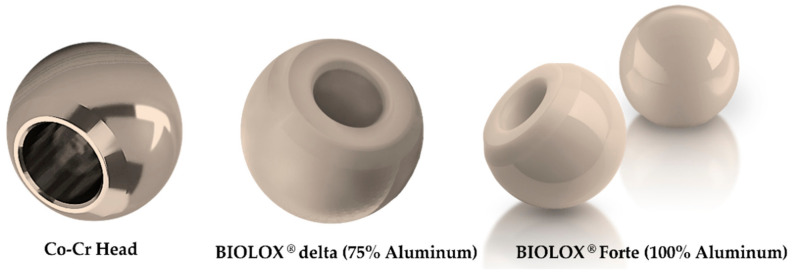
Acetabular heads of different biomaterials and brands [38].

Despite the outstanding mechanical properties of the cobalt-chromium alloy, a higher discrepancy of the Young’s modulus between the bone and the implant material causes stress shielding, improper micromotion at the bone–implant interface, and wear propagation from the acetabular cup junction. The ductileness of the implant body is decreased, and phase stability and alloy strength are increased by adding Ni, C, and N [37]. Another Co-Ni-Cr-Mo alloy, which contains 35% Co and 35% Ni is designed for it to have a high degree of corrosion resistance [37]. The wear debris from the implants may not be discharged through the urine, which can harm the human’s biological function and their immune system [16]. Different prominent manufacturers produce acetabular heads employing the cobalt alloys due to them having a wear resistance property. Figure 3 depicts the standard cobalt-chromium, Biolox delta, and Biolox forte acetabular head systems [38]. Biolox Delta has greater strength magnitudes and an excellent clinical record, with over 6 million more of these components having been implanted than the Biolox Forte one [39]. It should be mentioned that the Biolox Forte one (CeramTec, Plochingen, Germany) has not been in use for more than 10 years now because of them having fractures and squeaking.

#### 2.2.4. Ceramics Alloys

Ceramics are used in orthopedic implants because of their wear resistance, hardness, strength, and heat resistance properties [40]. Ceramics meet the most realistic hip implant joint demands. The ceramic joints’ performance varies based on the raw chemical compositions of them, and their production processes, process factors, injection procedures, and implant design. Alumina and zirconium are employed for making ceramics as bearing materials in orthopedic implant applications due to their tribological properties.

Alumina is an inorganic, nonmetallic material that is used in hip implants. Alpha alumina is the most used biomedical aluminum due to its high boiling point, rigidity, and low electrical and thermal conductivity. Alpha aluminum molecules are hexagonal and consist of Al^3+^ and O^2−^ ions, which form strong ionic and covalent chemical bonds, thereby resulting in a low electric and thermal conductivity and a high melting point. Alpha aluminum’s wettability makes it perfect for the implant joint bearings [41]. Having scratch resistance is important for decreasing the wear particle propagation, and alumina has higher degree of scratch resistance than other metal alloys do [42]. Therefore, it resists the wearing debris propagation [43,44]. Although alumina is preferred for the acetabular heads, the alumina stems should be avoided though since it has an extreme level of stiffness and a greater Young’s modulus. The brittle nature of alumina, which does not allow for plastic deformation, results in a massive amount of stress shielding at a fixed point of the acetabular head under in vivo loading conditions [42]. Additionally, any fracture that forms on the surface of the alumina particles develops rapidly because of them having a low fracture toughness value. The catastrophic fracture behavior of the alumina composites may be controlled by integrating a high-purity raw material with a smaller grain size and a greater density [16]. Researchers have invented a unique medical-grade alumina composite, Biolox^®^ [39]. In the modern acetabular cup modules, two aluminum composites are used: Biolox^®^ Forte (100% aluminum) and Biolox^®^ Delta (75% alumina and 25% yttria-stabilized tetragonal polycrystalline zirconia (Y-TZP)). Biolox^®^ Forte is the first biocompatible and chemically resistant Biolox^®^ aluminum composite [39]. In Biolox^®^ Delta, the zirconia atoms’ crystal structure transforms the fracture area from that which is metastable tetragonal to that which is stable monoclinic, thereby improving the degree of mechanical and fracture toughness and the strength of the alumina ceramics [45]. Figure 3 shows the Biolox Delta [33] and Biolox Forte [46] acetabular heads.

Due to its submicron-sized grains, a high degree of toughness, and crack resistance, zirconia is a desirable material for the implant [16]. Since it decreases the occurrence of catastrophic failures, zirconia-based oxide (ZrO_2_) is employed as a production material for the implant-bearing particles [47]. The unstable phase transition qualities of pure zirconia can be addressed by using a transformation toughening process, where the phase transformation can be stabilized during the manufacturing by adding MgO, CaO, or Y_2_O_3_ [48,49,50]. This method has been used to develop new zirconia additives for the orthopedic joints, including partly stable ZrO_2_, tetragonal zirconia polycrystals (TZP), and ZrO_2_-particle-toughened ceramics (ZTA). Zirconia-toughened alumina (ZTA), which is generated by hybridizing zirconia with alumina, has been employed in acetabular component manufacture since 2000 [51].

#### 2.2.5. Polymer

Due to Poly-tetra-fluoroethylene’s (PTFE) failure as an acetabular cup material, polymeric substance ultra-high molecular weight polyethene (UHMWPE) is used because of its high degree of wear resistance, ductility, and biocompatibility. The UHMWPE is a polyolefin that is made of long polyethene chains that are linked by the van der Waals force. The longer molecule chains and their overlap attract the neighboring chain forces, thereby reinforcing the molecular structure [52,53]. Each UHMWPE crystalline structure is linked by an amorphous zone [54].

**Figure 4:**
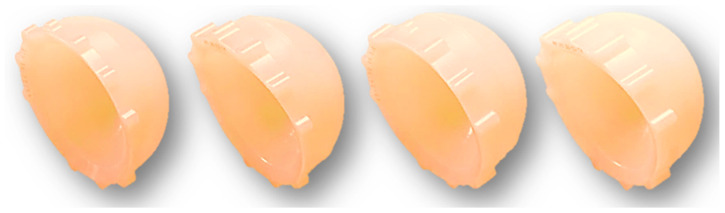
Different acetabular liner model designs with moderately cross-linked UHMWPE [61].

The crystalline structure of UHMWPE is affected by the temperature. Small crystalline melts at 60° to 70 °C, while bulk crystalline melts at 130° to 137 °C [55]. Although the number of molecules influences the mechanical properties of the UHMWPE, there being a greater number of molecules increases the elastic modulus of it, but it does not resist the wear rate of it [19]. Biocompatible UHMWPE has been used for the acetabular cup material, but the wear debris remains a problem. Annealing below the polymer’s peak melting point eliminates the free radicals that are inside the crystalline zone, and re-melting reduces the number of free radicals [56]. Re-melting the polymer after its irradiation reduces its crystallinity, thereby reducing its mechanical and fatigue properties [57]. Intermixing vitamin E with the polymer improves the oxidative stability of the highly cross-linked UHMWPE [58,59]. However, vitamin E reduces the cross-linking effectiveness, but intermixing and diffusion can mitigate this [60]. So, researchers are studying UHMWPE’s wear resistance, ductility, and biocompatibility to prevent the acetabular heads from loosening. Figure 4 shows KYOCERA Corporation’s UHMWPE acetabular liner models [61].

#### 2.2.6. Polyether-Ether-Ketone and Hydroxyapatite

Polyether-ether-ketone (PEEK) is a good mechanical material for the acetabular liner. PEEK’s biocompatible mechanical properties include a high melting point of 330–334 °C. It has biocompatible properties like titanium does that reduce the wear debris propagation [62] and can maintain sterility even when it is exposed to steam, radiation, or ethylene oxide [63].

Wang et al. [64] have reported that the PEEK-based acetabular components transmit the wear debris at half the rate of the UHMWPE/metal or UHMWPE/ceramic components. Pure PEEK, ternary, and binary PEEK composites are used in hip, spine, joint, and trauma implants. Carbon Fiber/PEEK is an FDA-approved binary PEEK composite with a higher Young’s modulus than pure PEEK has but that which is lower than the metal alloys. The CF/PEEK acetabular liner shells can withstand more stress than the binary PEEK composites can. Biological passivity, bone blindness, and lipophilicity in the human immune system limit its use in orthopedic implants [65,66]. BaSo4 is mixed into pure PEEK to make X-ray and MRI-compatible binary PEEK [67].

**Figure 5:**
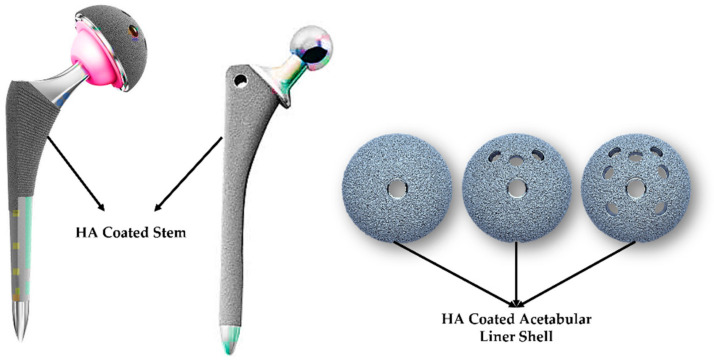
HA-coated hip stem and acetabular liner shell [33,61].

Hydroxyapatite (HA) is a calcium phosphate with the same shape and chemical composition as that of human bone. It has the same hexagonal shape and Ca/P ratio (1.67) [68]. An HA composite is used to coat the hip prostheses to promote the bone–implant integration [33,34]. The HA/PEEK composites have better osteointegration than the UHMWPE and pure PEEK ones do [67]. Ma et al. [67] have summarized the other PEEK composites that are made with carbon fiber, hydroxyapatite, poly (lactide), and tricalcium phosphate for best biological and osteointegration activity in bone growth and biological adhesion, respectively. The HA/PEEK composites with high-stress loading values are still being researched [69,70]. Figure 5 shows the HA-coated hip implant components [33,61].

The above discussion summarizes the mechanical and chemical properties of the metal, ceramic, and polyethene hip implants. The mechanical and chemical biomaterial properties of them are essential for the FEM analysis and in vitro implant validation. The hip implant materials must have a long fatigue life, favorable tissue reactivity, a low wear rate, and low bone resorption properties. An implant with a favorable mechanical and chemical profile will not always work better; the experimental data and specifications must be used to verify the results.

## 3. Performance Study of Hip Implant with Different Biomaterials in Pre-Clinical Stage

The FEM study of a hip implant with different biomaterials is essential for advancing the design of the hip implant system. Therefore, orthopedic researchers use the FEA methods to predict the implants’ performance parameters and to optimize the design of them. Researchers also use the FEM to analyze the failure mechanisms of the revised implants [71]. This section will describe the hip implant performance with different biomaterials and highlight the recent pre-clinical research.

**Figure 6:**
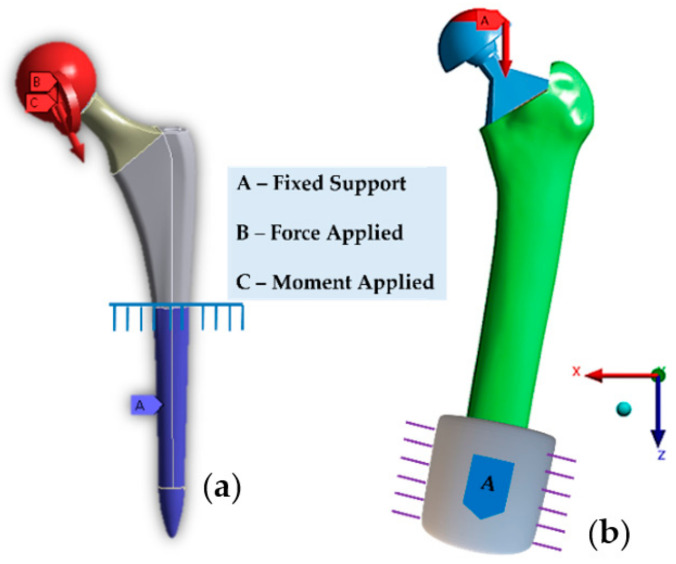
Finite element model view of (**a**) hip implant and a (**b**) hip implant with femur bone with ISO 7206-4 and ISO-7206-8 boundary conditions, respectively.

The FEA method can evaluate any physical phenomenon. Researchers use Ansys (Ansys Inc., Canonsburg, PA, USA) and Abaqus (ABAQUS Inc., Providence, RI, USA) FEM software to reduce the amount of manual testing and trials that are conducted and to standardize the design elements to improve the production efficiency. The FEM helps to determine the stress, strain, deformation, and fatigue life of an implant based on human activities. The preclinical performance of hip implants using an FEM is discussed in the next section. Figure 6a,b show an example the finite element model for a hip implant and implant that is integrated with the bone in the Ansys simulation software interface.

Von Mises stress/strain is a function of total stress/strain on an object’s body, which comprises normal stress/strain in the *x* and *y* axis along with shear stress/strain. The von Mises stress $\sigma_{V}$ on an object is determined by Equation (4) [72,73], which is shown below:

(6) $$\sigma_{V}=\sqrt{\left\{\sigma_{x}^{2}-\left(\sigma_{x}\times \sigma_{y}\right)+\sigma_{y}^{2}+\left(3\times T_{xy}^{2}\right)\right\}}\;\;\;\;$$

where $\sigma_{x}$ and $\sigma_{y}$ are the normal stresses in the *x* and *y* axes, respectively, and $T_{xy}$ is the shear stress in the *x*-axis.

Additionally, the equivalent von Mises strain, ${Ɛ}_{eq}$ on an object is determined by Equation (5) [73]:

(7) $${Ɛ}_{eq}=\frac{1}{\sqrt{2\left(1+V\right)}}\left[{\left({Ɛ}_{x}-{Ɛ}_{y}\right)}^{2}+{\left({Ɛ}_{y}-{Ɛ}_{z}\right)}^{2}+{\left({Ɛ}_{z}-{Ɛ}_{x}\right)}^{2}\right]\;\;\;\;$$

where ${Ɛ}_{x}$, ${Ɛ}_{y}$, and ${Ɛ}_{z}$ are strains in the *x*, *y*, and *z* axes, respectively. V is the poison ratio of the material.

Regardless of the theoretical mechanical properties of the various material alloys, the potential mechanical behavior of the hip implant should be determined by an FEM analysis. The von Mises stress and strain are very important to validate the pre-clinical implant behaviors. Table 2 compares the von Mises stress, strain, and deformation for several alloys [8,74,75,76,77]. Joshi et al. [74] have studied the hip implant’s mechanical performance (stress, strain, and deformation) under walking loading. The maximum mechanical properties across the walking load cycle show that the Co-Cr-Mo implant required the most stress to distort it the least, while the Ti-6AL-4V implant deformed significantly under a lesser amount of stress. Joshi et al. [76] have evaluated the mechanical behavior of Charnley’s hip implant under 2.3 kN, or that which is 3.5 times the typical body weight. The Co-Cr-Mo implants required the most stress to deform them the least, whereas other materials deformed substantially under a lesser amount of stress. The Co–Cr implant material expended far more stress with it having less deformation than Ti-4Al-6V did, as shown in Table 2. This was true for all of the implant forms and profiles. The Ti-6Al-4V implants [77] and the stainless steel implants [8] were stiffer because they consumed the maximum amount of stress in a less deformable state. The Co-Cr-Mo, Ti-6AL-4V, and the stainless steel which were stated in [17,23,24,25], consumed more stress to achieve the least deformation and strain due to their greater modulus of elasticity (E).

Stress shielding occurs due to there being large Young’s modulus mismatches between the implant and the bone, causing bone resorption and implant loosening to occur [78]. Anguiano-Sanchez et al. [79] have advocated covering the implant stem with PEEK to enhance the bone stress distribution. The coating stem improved the stress distribution over the cancellous bone by 85–90%. Darwich et al. [25] have examined the influence of the coating materials on the hip implant’s fatigue behavior, and they found that there was a 4.6% increase in the stress distribution for the coated hip stems over that of the un-coated implant stems. Enab et al. [80] examined the stress distribution over the femur bone for first and second-generation titanium alloys. The second-generation alloys had a lower Young’s modulus. The second-generation titanium alloys improved the stress distribution over the cancellous and cortical bone. Additionally, the HA-coated hip implants improved the stress distribution in all of the alloys. Yamako et al. [81] have used a finite element analysis to study the stress distribution and the bone mineral density over 10 years for Ti-6Al-4V and Ti-33.6Nb-4Sn. A hip implant that is manufactured from beta titanium alloy Ti-33.6 Nb-4Sn has 42.6% a higher bone mineral density than one that is created from alpha titanium alloy Ti-6Al-4V.

Reducing the implant’s rigidity indefinitely causes interface delamination and hinders the implant–bone connection, thus leading to the implant loosening [82]. A reduced implant stiffness may promote micromotion in the bone–implant contact area. Optimizing the implant material stiffness for in vivo loading circumstances requires addressing the micromotion and the contact pressure in the acetabular cup. Even though the above-mentioned FEM parameters can provide insight into the implant’s mechanical characteristics, additional computational FEM analyses, such as that of the micromotion, fatigue life estimations, safety factor estimations, damage percentage estimations, and wear estimations, are necessary to finalize the implant material due to the conflicting mechanical hypotheses about material stiffness.

### 3.1. Micromotion

Micromotion is bone–implant micro-movement that occurs under in vivo human loading [83]. It is crucial for the implant’s stability in the femur bone. The initial stability of it is established by it having 150–200 µm of micromotion at the bone–stem interface [84]. Micromotion that is above the prescribed limit may impede the bone’s in-growth with the stem, thereby causing aseptic implant loosening to occur. Since micromotion at the bone–implant interface depends on the implant load, the implant’s stiffness is critical [85]. This subsection summarizes the investigations that are related to hip implant micromotion.

Abdul et al. [84] have studied bone–implant micromotion for three implant materials. The composite alloys have the highest degree of micromotion due to their lower rigidity than Co-Cr and Ti-6Al-4V do. The Co-Cr and Ti-6Al-4V hip implants performed micromotions that exceeding 50 µm over 10%, 23%, and 51% of their surfaces. Otani et al. [85] have examined the micromotion of a cementless carbon composite, a titanium alloy, and a stainless-steel hip implant (200 GPa). According to [84], the hip implants that were made of carbon composite, which is a less stiff material, created more micromotion than implants that were made of other two stiff biomaterials did. In [86,87,88], the hip implants that were made of flexible and stiffer biomaterials were compared to quantify their micromotion. Flexible stems can stimulate aseptic infection and implant failure due to increased micromotion. Chen et al. [89] have used Anatomique Benoist Gerard (ABG) and Versys hip implants to study their micromotion and cancellous bone stiffness. When the cancellous bone density dropped from 100% to 50%, the micromotion that occurred rose to 150 µm. The micromotion ranges averaged 50 µm over 52% of the surface and 150 µm over 0.6% of the surface.

**Figure 7:**
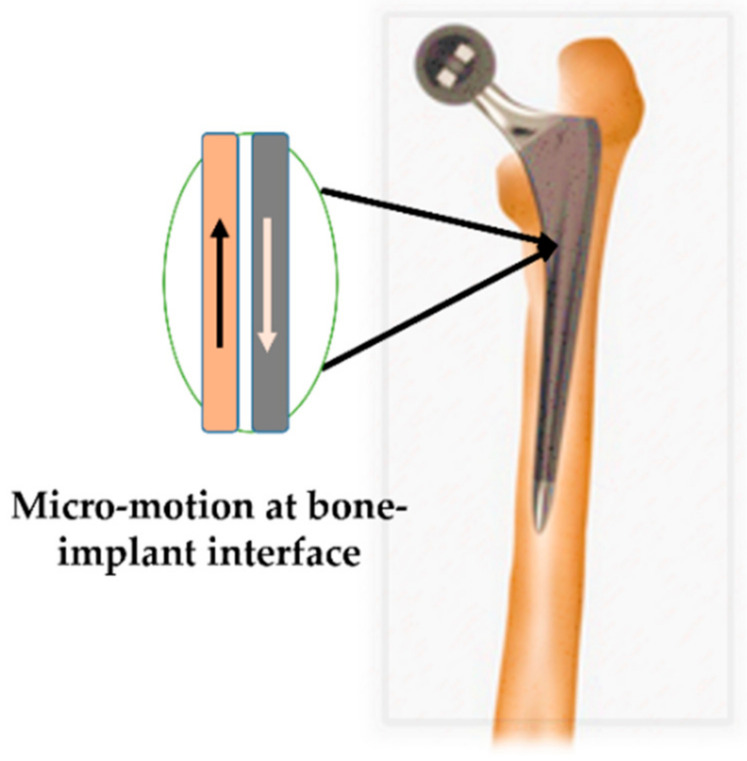
A typical bone–implant system with the micromotion region of interest is highlighted.

A stiffer implant material mismatches with the bone, resulting in poor load transfer and limiting the micromotion at the implant–bone interface [85]. Optimizing the implant material’s stiffness affects the bone–implant micromotion. The implant’s stiffness must be within the limits for proper micromotion and bone growth. Figure 7 shows a typical bone–implant system with the micro-motion highlighted.

### 3.2. Wear Estimation

Wear is a progressive interface degradation process that occurs at the implanted joint [90]. Wear debris contributes to the hip implant loosening due to there being joint surface defects, unfavorable soft-tissue responses [91], and foreign-body granuloma development [92], which can cause osteolysis and immobility [93]. The circular surface of the acetabular head is the main source of wear debris [94,95,96,97,98,99]. The head trunnion taper junction generates wear particles as is shown in recent works [92,100,101]. This section has two parts on the wear estimations. In this section, the wear generation properties of various materials that have been used in hip implant joints are summarized.

The wear volume is significantly dependent on the implant’s mechanical properties, including the contact pressure, wear coefficient, frictional coefficient, force, and sliding distance according to the wear estimation theories [19]. Since these factors are all functions of the material properties, the wear at the implanted joint is also dependent on the material properties. To optimize the material features of the hip implant joint, research is being conducted to estimate the volume of the wear in each material. A detailed analysis of this is given in the below subsections.

**Figure 8:**
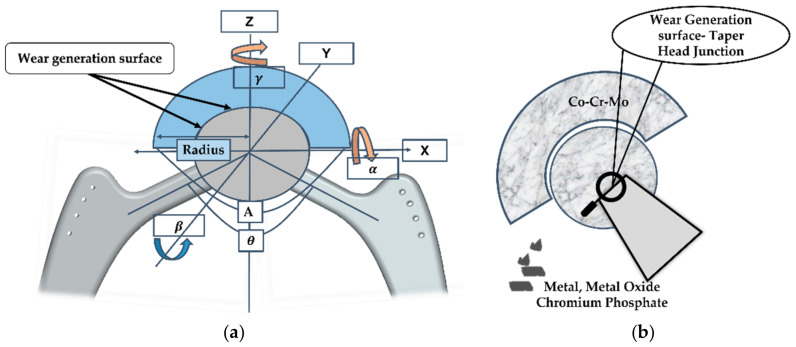
(**a**) Cross-sectional view of acetabular head–liner contact joint and (**b**) acetabular head—neck contact joint.

Figure 8 depicts a cross-sectional view of the acetabular head–liner contact joint and acetabular head–neck contact joint since those are the region of interest for a hip implant for wear generation [9,19]. Figure 8a shows the internal/external γ, the abduction/adduction β, and the flexion/extension α angles of the Z, Y, and X axes, respectively.

#### 3.2.1. Wear Rate for Different Hip Implant Biomaterials

This subsection provides an analysis of the wear estimations at the taper-head and acetabular cup-liner interfaces.

#### 3.2.2. Taper-Head Junction

Micromotion at the contact-pressured interface causes fretting corrosion to occur at the head–neck taper union. Due to the influence of the mechanical parameters on the wear, researchers use a finite element analysis to evaluate the taper–union junction wear during the pre-clinical phase. The angular misfit, contact surface area, head size, loading technique, surface roughness, and material combination affect the taper junction wear. This section highlights the impact of several material combinations on the head–neck taper junction wear.

Kyomoto [102] has evaluated the taper-head junction wear under 2 kN load for 5 million load cycles for Co-Cr and ZTA with a Ti-6Al-4V hip stem neck. ZTA-Ti-6Al-4V had a 1 mm^3^ wear rate, while Co-Cr-Ti-6Al-4V had a 3 mm^3^ wear rate. The authors found that the ZTA’s higher stiffness counteracts the relative micromotion at the taper junction, thereby lowering the wear rate. Morlock et al. [103] have used finite element analysis to evaluate the micromotion at the taper junction for a Co-Cr acetabular head and Co-Cr, Stainless Steel 316, and Ti-6Al-4V stems. Compared to the stronger stem materials, the Co-Cr-TMZF micromotion was the greatest at the taper junction. Fallahnezhad et al. [104] have studied the contact lengths and pressures in the Co-Cr/Co-Cr and Co-Cr/Ti-6Al-4V taper junctions. Co-Cr/Co-Cr produced a larger contact length and pressure than Co-Cr/Ti-6Al-4V did. Co-Cr/Co-Cr has stiffer properties that restrict the micromotion, hence it should create less micromotion than Co-Cr/Ti-6Al-4V would. Jauch et al. [105] have detected an increased amount of micromotion for the Co-Cr/Titanium head–neck material combination. Additionally, Michael et al. in [103] found that when it was compared to the CoCr-186 alloy, the Ti-47-12Mo-6Zr-2Fe (TMZF) alloy exhibited a considerably greater amount of micromotion. Haschke et al. [106] have found that replacing the trunnion’s material from Ti6Al4V with a softer material (Ti_12_Mo_6_Zr_2_Fe) accelerated the micromotions. Similar investigations [107,108,109] have indicated that the stiffer material combination for the head–neck of a hip implant inhibits the micromotion at the joint interface, but the mixed material combination with a higher–lower stiffness behavior was prone to fretting corrosion. According to wear estimation theory in [19] and [20], micromotion also known as the sliding distance, and it is the most important factor in the development of wear. Therefore, it can be stated that a softer material combination increased the micromotion that accelerates the rate of wear generation in comparison to that of stiffer materials.

#### 3.2.3. Acetabular Head–Liner Junction

**Figure 9:**
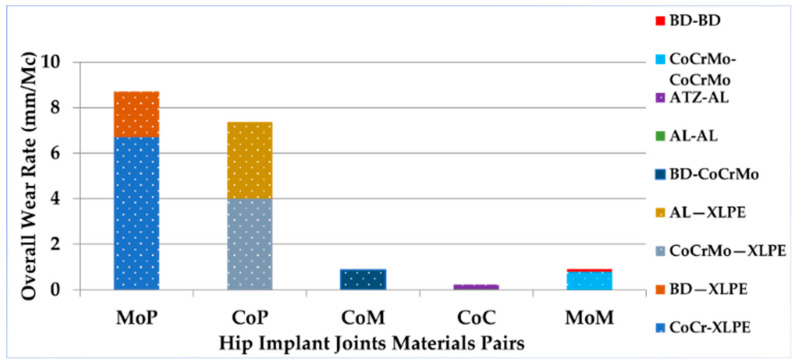
An illustration of wear rate/million cycles for different hip implant joint material pairs.

The acetabular head–liner interface wears owing to the multi-directional head movement that is produced by human activities. As the most mobile joint in a hip implant system, the acetabular–liner interface must be optimized by evaluating the wear at this biomaterial interface. According to the market research, the acetabular head–liner junctions are either hard-on-hard (HoH) or hard-on-soft (HoS) ones. There are four varieties of HoH bearings: ceramic-on-ceramic (CoC), ceramic-on-metal (CoM), metal-on-metal (MoM), and metal-on-ceramic (MoC). The HoS bearings come in two types: metal-on-polyethene (MoP) and ceramic-on-polyethene (CoP). The MoP implant joint material pairings had the highest overall wear rate (mm^3^/Million Cycles), while CoC had the lowest overall wear rate, as shown in Figure 9.

Uddin et al. [110] have studied the wear rate for three material combinations: polycrystalline diamond (PCD) on PCD (975 GPa), ceramic-on-ceramic (375 GPa), and metal-on-metal (210 GPa). The MoM one had the highest wear rate after 2 million walking load cycles, while the PCD-on-PCD one had the lowest wear rate due to it having a lower wear coefficient. Jamari et al. [111] have examined the contact pressure at the acetabular head–liner interface with three metal-on-polyethylene material combinations in response to the walking gait cycle: Ti-6Al-4V (110 GPa)-on-UHMWPE (1.4 GPa), Co-Cr-Mo (210 GPa)-on-UHMWPE, and (SS 316L) (193 GPa)-on-(UHMWPE) (UHMWPE). The (Ti-6Al-4V) (110 GPa)-on-(UHMWPE) one (1.4 GPa) had the lowest contact pressure for the acetabular head–liner junction. The same author, Jamari et al. [112], evaluated the contact pressure for three metal-on-metal acetabular headliner material combinations: Ti6Al4V-on-Ti6Al4V, CoCrMo-on-CoCrMo, and CoCrMo-on-Ti6Al4V (SS 316L-on-SS 316L). The Ti6Al4V-on-Ti6Al4V one reportedly had the lowest contact pressure. Since contact pressure is linked to the wearing volume rate, the lowest available material combinations will yield the lowest wear rate.

The hard-on-soft bearings (MoP or CoP) have a higher wear rate (mm^3^/million cycles) than the hard-on-hard bearings do (ceramic-on-ceramic (CoC) and metal-on-metal) (MoM). According to the biomaterials properties, the implant joints with a large variation in the Young’s modulus absorb more stress and impose a larger contact pressure on an acetabular cup with a lower degree of stiffness. A greater amount of contact pressure exceeds the ultimate stress strength of the less stiff materials, such as a polyethene or a cross-linked polyethene acetabular cup, thereby causing damage to the cup’s contact area when it is paired with a PCD acetabular head. If the contact pressure is within the limit of the acetabular cup-liner junction’s ultimate stress strength (for CoC and MoM), it results in less damage occurring to the contact surface area along with a lower wear rate. The MoM implant junctions exhibited a lower wear coefficient. The metallic and ceramic wear debris from the hard material joints can cause tissue injury and osteolysis, which are a major research concern.

### 3.3. Fatigue Behavior Analysis

Estimating the fatigue life of an artificial hip implant for various material combinations under the most typical loading scenarios is a key criterion for predicting the functional life and the mechanical characteristics of them. Fatigue arises in an object’s body in three stages: crack initiation, crack growth, and fracture reaction to repetitive cyclic stresses [113]. Shear stress and strain energy cause tiny extrusions and surface damage owing to effect of the back-and-forth stress. This subsection describes the fatigue behavior analysis of the hip implant systems with different biomaterials.

#### 3.3.1. Fatigue Life Estimation for Fully Reversed Loading Conditions

**Figure 10:**
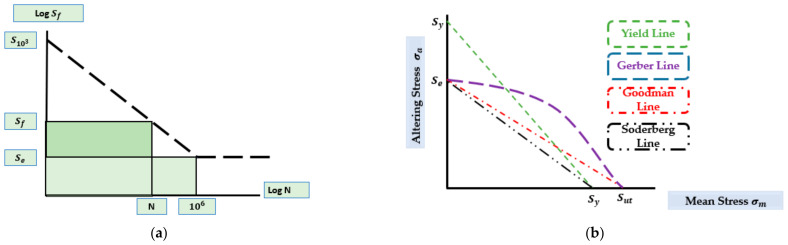
(**a**) S-N diagram following industry standards for high-cycle loading, (**b**) graphical representation of fatigue estimation models of non-fully reversed loading conditions.

The S-N diagram approach with a log–log scale is the most frequently used method for estimating the fatigue life in the case of fully reversed loading conditions, where the fatigue life boundaries are within $10^{3}<N<10^{5}$ cycles. The S-N diagram in Figure 10a is defined following the industry standards, where *S_f_* and *N* represent the fatigue strength and life, respectively, as indicated by the vertical y and horizontal x axes. Considering that the fatigue life *N* is related to the fatigue strength *S_f_*, it is preferable to keep the fatigue strength $S_{f}$ equal to or below the endurance strength level $S_{e}$ so that the component can obtain a fatigue value that is greater than 10^6^ cycles or the infinite range, respectively. Here, $S_{10^{3}}$ is the lowest fatigue fatigue strength limit in terms of the lowest fatigue life. The fatigue life N is computed using the Formula (6), assuming that the maximum contact stress $\sigma_{max}$ is contained within the range $\left(S_{10^{3}}<\sigma_{max}<S_{e}\right)$ MPa [114].

(8) $$\;\;N={\left(10^{-\bar{C}}*\sigma_{max}\right)}^{\frac{1}{b_{s}}}\;\;\;\;$$

where $b_{s}$ dentoes the slope in the S-N diagram, and $\bar{C}$ denotes the intersection of this slope with the y axis in the S-N diagram. Hence, $\bar{C}$ and $b_{s}$ are defined by using the below Equations (7) and (8).

(9) $$\;\;\;\;\;\bar{C}=Log\frac{{\left(S_{10^{3}}\right)}^{2}\;\;\;}{S_{e}}\;\;\;\;$$

(10) $$\;\;\;\;\;b_{s}=-\frac{1}{3}log\frac{S_{10^{3}}}{S_{e}}\;\;\;\;\;\;$$

Furthermore, estimating the allowable stress that is required to prolong the life of the fatigue factor is shown in Equation (9).

(11) $$\;\;\;\sigma_{allow}=\frac{S_{f}}{n}\;\;$$

where $S_{f}$ and *n* denote the material’s fatigue strength and the component’s safety factor, respectively. However, it is worth noting that the aforementioned equation indicates that the estimation of the fatigue life is highly dependent on the contact stress. Since the loading conditions that are applied to the acetabular head influence the contact stress, the load amplitude must be precise.

#### 3.3.2. Fatigue Life Estimation Using Non-Fully Reversed Loading Conditions

Once the mean stress is zero, the fully reversed cyclic loading conditions based on the S-N diagram can forecast the fatigue life. The traditional S-N diagram method must be adjusted when the mean stress is not zero. Figure 10b displays the fatigue estimate models of the non-fully reversed loading scenarios with a non-zero mean stress. Four boundary lines for the Goodman, Gerber, Soderberg values, and the yield estimate the safe and risky zones. The lower and upper lines correspond to safe and unsafe zones for each component, respectively. The components within and above the safe zone have fatigue lives that are above and below 10^6^ cycles, respectively. The Soderbergh and yield lines relate to the safe and unsafe zones by connecting the endurance and the yield stress points. Both the Goodman and Gerber’s lines tie the yield stress points with the endurance stress to the safe and risky zones, respectively. The fatigue safety factor *N_f_* which is linked with mean stress *σ_m_* and the changing stress *σ_a_* can be determined using Equation (10) [114].

(12) $$\;\;\;\;\;\frac{\sigma_{a}}{S_{e}}+\frac{\sigma_{m}}{S_{y}}=\frac{1}{N_{f}}\;\;\;$$

Here, $S_{e}$, $S_{y},\;\mathrm{and}$ $S_{ut}$ are the endurance limit and the ultimate tensile stress, respectively. Equation (11) defines the equivalent altering stress for the Goodman and Soderberg fatigue life estimation models, respectively. Additionally, the implant’s fatigue life is estimated using the Equation (12).

(13) $$\;\;\;\sigma_{A}=\frac{S_{y}\times \sigma_{a}}{S_{y}-\sigma_{m}}\;\;\;\;\;\;$$

(14) $$\;\;\;\;N={\left(10^{-\bar{C}}\times \sigma_{A}\right)}^{\frac{1}{b_{s}}}\;\;\;\;\;$$

#### 3.3.3. Fatigue Life Estimation for Different Biomaterials

Senalp et al. [11] have tested four Ti-6Al-4V and Co-Cr hip implants under a 3 kN stress. The authors used the four hypotheses to calculate the fatigue safety factor, and they found that a Ti-6Al-4V hip implant was safer than a Co-Cr alloy implant was. Kayabasi et al. [115] have used an FEM to calculate the fatigue safety factor of the Ti-6Al-4V and Co-Cr hip implants. The Ti-6Al-4V implants had a greater safety factor, which is in agreement with the prior study.

Considering the mathematical analysis of the above fatigue life calculation methods, the fatigue life N is dependent on the artificial hip implant component’s stiffness. Equation (12) shows that the effective modifying stress increases as the material tensile and yield stresses rise. A stiffer material with a higher ultimate tensile stress value increases the effective alteration stress and the maximal contact stress, which increases the implant’s fatigue life. A reduced S_y_ value results in a lower effective altering stress, which compensates for the fatigue life. The theoretical formula acknowledges the studies in [11] and [115], and it has showed that as Ti-6Al-4V has larger ultimate and yield strength, the fatigue safety factors were higher for the Ti-6Al-4V hip implant than they were for the Co-Cr hip implant. This information can be used to estimate the fatigue life cycle of the different biomaterials for the hip implants. This helps to estimate the quality and lifespan of the implant.

## 4. Biocompatibility and Tissue Response for Different Biomaterials in, In Vitro, In Vivo, and Clinical Stage Studies

Even though the computational finite element analysis showed the mechanical performance of different biomaterial hip implants, in vitro, in vivo, and clinical studies are still needed before its production to conducted. This part will provide insight into the hip implant’s response in, in vitro, in vivo, and clinical stages as the complications cannot be illustrated solely by a computational analysis.

### 4.1. In Vitro and In Vivo Studies

With there being several material compositions for the implants’ applications, it is important to validate the implant biomaterials’ safety by confirming their biocompatibility and understanding their reaction in the inflammatory tissue environments. Before clinical testing in the human body can be conducted, the biomaterials must undergo in vitro and in vivo trials to confirm their biocompatibility and tissue reaction [116].

Bich Vu et al. [117] have tested the biocompatibility of Ti-6Al-4V and UHMWPE in rabbits and mice for 12 weeks. This study found that there were no adverse effects on the muscle or the skin. After 12 weeks, the skin had no inflammatory cells, macrophages, aberrant cells, or collagen. Takamura et al. [118] have studied the carcinogenicity and chronic toxicity of 316L stainless steel, nickel, Ti-6A1-4V, hydroxyapatite (HA)-coated Ti-6A1-4V, an aluminum oxide containing yttrium oxide, and a zirconium oxide containing yttrium oxide in mice for 24 months. The nickel alloy was carcinogenic and toxic, but the other biomaterials were not. The effects of the HA particles on cell damage, apoptosis, and cytotoxicity were studied in vivo in [119]. The authors of this study reported that the 20–80 nm HA wear particles’ triggers were found to be responsible for triggering the apoptosis and cytotoxicity. One hundred-to-two hundred-micron HA wear particles did not generate any chromosomal abnormalities in the rabbits [119].

### 4.2. Clinical Stage

The biocompatibility and tissue response investigation in the clinical stage may be divided into two sections: the bone remodeling and the hip implant’s survival rate with different biomaterials and the wear debris complications. Bone remodeling at the interface between the implant and the bone is a significant component of the hip implant system that determines the mechanical stability and fatigue life of the implant. Due to adverse tissue reactivity to the wear debris that is generated by the implant joint, the material combination selection for the hip implant joint’s components is critical. Even though the rate of the joint wear can be reduced by deploying tougher biomaterials, adverse local tissue response analyses are still necessary to establish the biocompatibility of the hip implant joint’s material.

#### 4.2.1. Impact of Bone Remodeling and Coating of the Hip Implant with Different Biomaterials

The load transfer and stress shielding properties of the hip implant stem have a significant impact on the bone remodeling following a THR. Therefore, the material properties of the hip implant system are intimately related to bone remodeling. The theoretical study suggests that more stiff biomaterials cushion the stress, which eventually slows the rate of bone remodeling.

Four different types of hip implants including the CLS Spotorno (Ti-6Al-4V stem), Vision 2000 (Co-Cr-Mo Stem), Alpha-Fit (Ti-6Al-4V stem), and mayo (Ti-6Al-4V stem) were the subject of a bone remodeling follow-up study that was conducted by Steffen Brodt et al. [120]. The recent research shows that the Vision 2000 had the greatest influence on the stress shielding because of the use of a stiffer Co-Cr-Mo material, which led to the greatest drop in the bone density values [120]. Five different hip implant types—the CLS Spotorno (Ti-6Al-4V stem), the AML (Co-Cr-Mo Stem), the Corail (Ti-6Al-4V stem), the Taperloc (Ti-6Al-4V stem), and the classic version (Ti-6Al-4V)—were used in a follow-up study on bone remodeling by Charles Rivière et al. [121]. In contrast, the AML outperformed the other hip stem systems in terms of the stress shielding and the bone remodeling rate. Craven TG et al. [122] have found that a stiffer orthopedic implant produces a higher rate of bone mineral loss in the vicinity of the implant.

Yun-Lin Chen et al. [123] have compared the use of a hydroxyapatite coating and a porous structure in the hip implant systems. It was reported that an HA-coated hip implants outperformed the porous coatings in terms of the proximal femoral osteointegration, the preservation of the peri-prosthetic bone quality, the stimulation of the bone growth, the biological fixation with the bone, and its mechanical stability. Svehla et al. [124] have investigated the impact of the hydroxyapatite (HA) coating thickness on the bone formation and the shear strength in a sheep model. A one-hundred-µm-thick HA layer showed improved to the adhesion and the on-growth element as well as it having less degradation when it was compared to the 50 µm thick layer.

#### 4.2.2. Impact of Wear Debris on the Hip Implant System

The patients’ health and quality of life are negatively affected by the wear debris from the implant joints. At least 10% of the hip implant patients with the metal-on-metal (MoM) hip implants and a lesser percentage of the patients with the metal-on-polyethylene (MoP) hip implants experience an implant failure per year due to the adverse tissue response that occurs due to the metal wear debris. This subsection illustrates the various types of wear debris from different implant joints, as well as the adverse tissue responses to wearing debris.

#### 4.2.3. Taper Junction and Acetabular Head–Liner Junction

**Figure 11:**
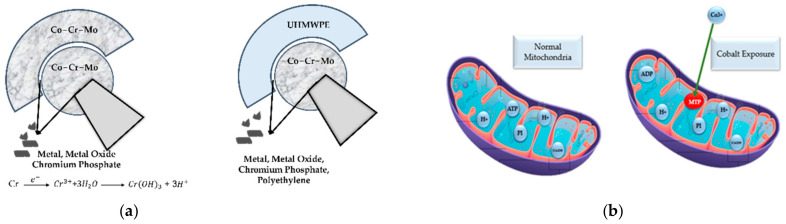
(**a**) Different wear debris generation and associated chemical reactions at hip implant joints, and (**b**) presence of cobalt ions in mitochondria.

A Ti-6Al-4V and Co-Cr-Mo material combination for a hip implant’s stem and acetabular head creates Cr^3+^ and Ti+ wear debris [125,126]. Cr^3+^ wear debris reacts with the synovial fluid to form Cr(OH)_3_ and H+. According to a clinical trial in [127], the MoP hip implant fretting taper junction’s wear debris are chromium and phosphate-rich particles. The wear debris of the Cr^3+^ and low-Co ions come from the MoM head–liner connections, while the oxide particles of the Cr, low-Co, and sub-micron metallic particles come from the MoP junctions. Figure 11a shows the wear and synovial fluid interaction of this. The wear debris are 300 nm in size for the polyethene ones, 90 nm in size for the metal ones, and 30 nm in size for the ceramic ones [128]. The clinical research [129] shows that the MoM material combinations generate 80% less metal wear than the MoP material combinations do. Metal wear debris destroys the human tissues, despite there being an decreased MoM wear rate. Similar research shows that in [130,131], the hip implant acetabular head joints include four forms of wear droplet particles: chromium oxide, Co-Cr-Mo metal debris, chromium phosphate, and chromium III oxide, whereas the Co-Cr-Mo wear debris dominates the acetabular head material relative to the MoM one.

According to a clinical study [132], the metal ions from fretting and articulating the joints cause more tissue damage than the metal debris does. The joint metal debris reacts with the lysosomes in the synovial fluid, releasing metal ions [133]. According to [134], Co-Cr-Mo alloys generate 13:2:1 more ions than other metals do.

#### 4.2.4. Adverse Tissue Response to Wear Debris and Ions

Papageorgiou et al. in [135], employed dermal fibroblasts to investigate the tissue damage in a Co-Cr-Mo alloy, and they found that the nano-sized Cr^3+^ wear generated more ion release, which in turn caused a higher rate of tissue damage than the micron-sized wear debris did. In addition, the cobalt ions are more hazardous than the particles are since they generate reactive oxygen species that damage the lung fibroblasts [136] and the T cells [137]. Although the early research in [138] indicated that the majority of the lesions and cell damage are the results of an excessive wear rate from the hip implant joint. The recent studies in [139] have shown that MoP and MoM hip implants have a shorter life span, despite them achieving a lower wear rate concentration in human tissues.

The pathological stability of the cobalt ions in the cell system induces mitochondrial permeability transition pores (MPT) in mitochondria, as depicted in Figure 11b. Additionally, the inclusion of MPT increases the permeability of the mitochondrial internal membrane, resulting in a reduced electrochemical link between the inter-membrane space and the mitochondrial matrix [126]. Subsequently, the rate of adenosine triphosphate (ATP) generation is reduced due to a decrease in the proton motion force, which further promotes a hypoxia response activation. The conclusion of this MPT inclusion chain reaction was reported in [126] in the synovial inflammation and the macrophage-dominated lesions in the human tissue system.

However, the presence of ions, proteins, and proteoglycans alters the physicochemical behavior of the human tissue by dropping the pH and the oxidant levels [140]. Inflammation or hypoxia may also arise from a shift in the pH, owing to the augmented glycolytic routes and associated growth in the lactate or pyruvate synthesis [141]. López-López et al. in [142] examined a variety of clinical studies to evaluate the selection of the implant combos in total hip replacements, where the metal-on-polyethene, ceramic-on-polyethene, ceramic-on-ceramic, or metal-on-metal types were used. In addition, the study found that the metal-on-polyethene and ceramic-on-polyethene-based hip implants had a lower risk after 0 to 10 years compared to the other material combinations.

Martino et al. [143] have reported the survival rates in an investigation of six different types of hip implants with different material combinations, wherein it was found that the CoC, CoP, and MoP material combination-type hip implants had a greater survival rate than the MoM-type hip implants did. Moreover, it is noticeable from in vitro, in vivo, and clinical studies that the fatigue life or survival of the hip implant is not only dependent on the mechanical properties of biomaterials. The mechanical properties of the hip implant are just a part of the fatigue life estimation. The hip implant system needs to be biocompatible and free from adverse tissue reactions to obtain higher fatigue or survival life values.

## 5. Major Factors of THI with Different Biomaterials and Forthcoming Research Directions

Due to the issues with the current hip implant biomaterials, a study could not identify a specific material combination. Based on relevant studies, this section outlines the significant biomaterial parameters that affect the hip implant’s performance and life. This section also discusses the biomaterial research issues for the THI systems.

Recent clinical investigations have shown that the implant stem materials must be biocompatible, nontoxic, and biologically active to enable the bone–implant integration. A stronger hip implant can handle more stress with less distortion, which is needed to prevent the implant system fatigue cracks occurring. A hip implant that is manufactured from a material with a higher modulus of elasticity provides a significant amount of stress shielding, which damages the bone tissue. To circumvent this contradiction, a material must be designed that can withstand higher loading and have uniform stress distribution across the implant body, thereby preventing the stress shielding.

Implants loosen when the bone–implant’s range of micromotion is exceeded. The literature suggests that a stiffer material can reduce the implant’s micromotion, although this contradicts with the stress shielding requirement. A recent study showed [123] that HA coating to the implant’s body surface reduces the amount of micromotion and increases its mechanical stability. Estimating the HA coating thickness on the implant body is a work in progress because the coating thickness affects the implant–bone micromotion.

The wear debris from such joints cause the joints to loosen and unwanted tissue reactions in the body, therefore the hip implant joint’s material combination is also a concern. The hip implant joints are made up of hard-on-hard (CoC or MoM) and hard-on-soft (MoP or CoP) bearings. Hard-on-soft implant joints have a greater volume wear rate per million cycles. Researchers have developed many approaches like cross-linking the polythene ions with vitamin E and reinforcing the polyethene with carbon nanotubes to improve the corrosion resistance and mechanical properties of them while reducing the amount of wear. Hard-on-hard implant joints wear less than those that are composed of soft materials, but the metal wear particles are more dangerous and poisonous, and as such, they harm the key organs and provoke asthma attacks. Even though much research has been done to improve the implant joint materials, wear debris is still a key concern and a future area of research.

The longer fatigue life of the hip implants is related to their mechanical performance and biocompatibility in humans’ adverse tissue environments. Thus, a complete hip implant material selection must meet the pre-clinical, in vitro, in vivo, and clinical performance assessments.

## 6. Conclusions

This article focuses on the biomaterials and the associated performance matrix that is estimated in the THI system’s pre-clinical, in vivo, in vitro, and clinical stages. To the best of the author’s knowledge, this has not recently been documented as a review article. Following the research objective, the article was divided into three major sections. This article began by discussing the physical and chemical properties of the biomaterials that are used in hip implants. Second, this study addressed and evaluated the performance of the hip implants using those biomaterials in relation to the FEM computational analyses in the pre-clinical stage. The hip implant’s performance matrix is linked to the various biomaterial estimations in the pre-clinical stage, including von Mises stress, deformation, and micromotion at implant–bone contact, wear estimation at the hip implant joint, and the fatigue failure properties. Third, in vitro, in vivo, and clinical biocompatibility and tissue response studies of the hip implant with various biomaterials are described. Bone remodeling at the implant–bone contact surface as well as an adverse local tissue response to the wear debris that is generated by hip implant joints at all of the stages are discussed. Finally, the critical considerations and future research challenges in the optimal material combination selection for hip implant systems are summarized to reduce the risk of a system failure.

## Figures and Tables

**Table 1:** Material properties of commercially deployed biomaterial alloys in THRs [17,23,24,25].

Materials	Type	Young’s Modulus (Gpa)	Yield Strength (Mpa)	Ultimate Strength (Mpa)	Poisson Ratio	Density (Kg/m^3^)
Ti6Al4V	α + β	110	800	900	0.342	4500
Ti6Al7Nb	α + β	120	950	1050	0.33	4510
Ti-29Nb-13Ta-4.5Zr	β	40–80	>1000	911	0.33	5000
Ti-15Mo-5Zr	β	78	920	960	0.33	5060
Ti-15Mo-5Zr-3Al	β	82	864	1475	0.3	4950
Ti-13Nb-13Zr	Near β	84	900	1037	0.3	4990
Ti-29Nb-13Ta-4.6Zr	β	80	864	911	0.3	5000
Ti-35Nb-7Zr-5Ta	β	55	596	742	0.3	5000
Ti-13Nb-13Zr	β	82	908	1037	0.3	4990
Ti-35Nb-5Ta-7Zr-0.4O	β	66	976	1010	0.34	5600
Ni-Ti Alloys	-	58	472	1290	0.325	6560
Ni-Ta Alloys	-	83	690	895	0.3	6450
Inconel 718 (UNS N07718)	-	200	1100	1375	0.29	8230
Co-Cr	-	220	450	270	0.3	8500
Co-Ni-Cr-Mo	-	230	1000	1650	0.29	8700
Bio-steels	-	210	180–600	480–900	0.29	7500
UHMWPE	-	0.689	20.7	40	0.33	931–949
PEEK	-	3.76–3.95	87–95	-	0.37	1230
HA		13	38	48	0.27	3005

**Table 2:** Comparative Evaluation of von Mises stresses, strains, and deformations for various material alloys.

References	Implant Stem Material	Von Mises Stress (MPa)	Equivalent von Mises Strain	Deformation (mm)
Şensoy et al. [8]	Nickel–titanium alloy	980	0.01695	15.35
	Stainless steel	1104	0.00526	13.94
	Ti6AlV	989	0.008999	16.59
Joshi et al. [74]	Co-Cr-Mo	575	0.0028	0.155
	Ti-6AL-4V	550.00	0.0055	0.35
	Ti-6Al-7Nb	540.00	0.0049	0.33
Chethan et al. [75]	Ti–4Al–6V	622.24	0.0054	0.490
	Co-Cr	623.48	0.0031	0.28
Joshi et al. [76]	Ti-6AL-4V	622.24	0.01	0.49
	Co-Cr Alloy	722.7	0.0039707	0.25684
	Co-Cr-Mo	728.88	0.003417	0.21916
	Ti-6Al-7Nb	702.75	0.0062826	0.41773
	Ti-6Al-4V	709.64	0.0069297	0.45639
	Ti-29Nb-13Ta-4.6Zr	722.7	0.0097282	0.62927
	Ti-13Nb-13Zr	722.7	0.009265	0.5993
Kumar et al. [77]	Ti-6Al-4V	0.58	5.27 × 10^−6^	0.45
	Ti-6Al-7Nb	0.575	4.7 × 10^−6^	0.43
	Ti-13Nb-13Zr	0.583	7.07 × 10^−6^	0.58

## Data Availability

Not applicable.
